# Thermosensitive Poloxamer-*graft*-Carboxymethyl Pullulan: A Potential Injectable Hydrogel for Drug Delivery

**DOI:** 10.3390/polym13183025

**Published:** 2021-09-07

**Authors:** Marieta Constantin, Bogdan Cosman, Maria Bercea, Gabriela-Liliana Ailiesei, Gheorghe Fundueanu

**Affiliations:** “Petru Poni” Institute of Macromolecular Chemistry, 41-A Gr. Ghica Voda Alley, 700487 Iasi, Romania; cosman.bogdan@icmpp.ro (B.C.); bercea@icmpp.ro (M.B.); gdarvaru@icmpp.ro (G.-L.A.); ghefun@icmpp.ro (G.F.)

**Keywords:** pullulan, poloxamer 407, injectable copolymer, sol–gel transition, drug delivery system

## Abstract

A thermosensitive copolymer composed of amphiphilic triblock copolymer, poloxamer 407, grafted on hydrophilic pullulan with pendant carboxymethyl groups (CMP) was prepared and characterized. The structure of the new copolymer was assessed by Fourier transform infrared (FT-IR) and ^1^H nuclear magnetic resonance (^1^H NMR) spectroscopy. The content of the poloxamer in the grafted copolymer was 83.8% (*w*/*w*). The effect of the copolymer concentration on the gelation behavior was analyzed by the vertical method and rheological tests; the gel phase of the copolymer occurred at a lower concentration (11%, *w*/*v*) as compared with poloxamer (18%, *w*/*v*). The starting gelation time under the simulated physiological conditions (phosphate buffer with a pH of 7.4, at 37 °C) was sensitive on the rest temperature before the test, this being 990 s and 280 s after 24 h rest at 4 °C and 20 °C, respectively. The rheological tests evidenced a high elasticity and excellent ability of the copolymer to recover the initial structure after the removal of the applied force or external stimuli. Moreover, the hydrogel has proved a sustained release of amoxicillin (taken as a model drug) over 168 h. Taken together, the results clearly indicate that this copolymer can be used as an injectable hydrogel.

## 1. Introduction

Injectable hydrogels are attracting supports for drug delivery and tissue engineering applications due to their physical (easily manipulated) properties and minimally invasive delivery and adapting shape in real time [1,2]. The hydrogel precursor loaded with drugs and/or target cells can be injected at the wound site where it presents in situ sol–gel transition due to physical or chemical stimuli. Most importantly, injectable hydrogels have a microstructure similar to the extracellular matrix (ECM) and allow good physical integration into the defect, possibly avoiding an open surgical procedure and facilitating the use of minimally invasive approaches to the delivery of materials and cells [2]. Injectable hydrogels can be prepared by physical or chemical crosslinking of polymers, the mechanical properties of the latter ones being considerably improved [3,4]. Thermally induced gelling systems seem to be the most promising ones for the development of injectable drug delivery systems, their formulation being simple, totally free of organic solvent and offering the possibility of drugs solubilization prior to forming a gel matrix [5,6].

Amphiphilic random or block polymer chains that form a macroscopic gel (thermogel) at a certain temperature are successfully applied in cell therapy, tissue regeneration and wound healing due to their biocompatibility and long-lasting persistence in gel form in vivo [2]. Poloxamers (Pluronic^®^) are a family of triblock copolymers with a center block of hydrophobic polypropylene oxide (PPO) flanked by two blocks of hydrophilic polyethylene oxide (PEO) [7]. Among the series of Pluronics, the poloxamer 407, a nonionic surfactant with a wide range of applications, is known as an “inactive ingredient” by the U.S. Food and Drug Administration (FDA) for a variety of medicinal products, such as oral solutions, suspensions, inhalation formulations, intravenous (IV), ophthalmic or topical formulations [8,9]. It was found that injected Pluronic in rats is eliminated in a great measure within 3 days, especially by renal excretion and also, to a smaller extent, by biliary excretion [10]. Other studies demonstrated that doses up to 8.75 mL of 22% Pluronic F127 can be administrated to a 70 kg person without inducing hyperlipidemia or altering other blood values [11]. The concentrated aqueous solutions of poloxamer 407 become a thermoreversible gel, which makes this copolymer an interesting formulation choice for optimizing drug formulations and drug administration applications [12,13,14]. The transition temperature of poloxamer 407 from aqueous solution to gel (Tsol–gel) depends on the polymer concentration and increases with decreasing the poloxamer concentration. Below a critical concentration threshold, poloxamer no longer gels due to the low number of hydrophobic interactions between polymer chains [7]. Furthermore, the addition of pharmaceutically active ingredients, salts, excipients and other compounds to poloxamer 407-based formulations may increase or decrease the Tsol–gel [15,16].

The grafting of synthetic polymers on natural polysaccharides has been widely used as one of the most convenient ways to combine the advantages of natural and synthetic macromolecules, particularly in applications in which the interactions are desired to manipulate biological responses, such as growth factor binding or enzymatic degradation. [17]. The nonhydrolytically degradable block polymers are often conjugated to natural molecules to impart points of local hydrolytic or enzymatic degradation [18,19,20]. Compared to poloxamer copolymers, grafted copolymers can form reversible gels with significant elastic modulus in water at a lower concentration above a certain temperature. Recent studies have reported the synthesis and evaluation of poloxamer 407-grafted copolymers, i.e., poly(acrylic acid) [21,22,23,24], oligo(lactic acid) [25], chondroitin 6-sulfate [26], heparin [27,28], alginate [29,30,31], chitosan [32,33] and hyaluronic acid [34] as efficient vehicles for protein, vaccine and drug delivery using in situ formation property. Most of these gels were stable against degradation for a least 20–30 days. Poloxamer 407-grafted chitosan showed improved aqueous stability and biocompatibility, which was due to the polysaccharide moiety [33].

On the other hand, pullulan is a biopolymer produced of the yeast-like fungus *Aureobasidium pullulans* [35], being blood compatible, biodegradable and nontoxic, with no mutagenicity or carcinogenicity [36,37]. It is a nonionic polymer with good water solubility properties. The basic structure of the pullulan consists of maltotriose units linked by α-(1→6) bonds [38]. Pullulan has been derivatized into various forms that have significant biomedical applications [39,40]. Moreover, pullulan and its derivatives and its conjugation with other polymers and complex forms have special applications in tissue engineering [41], drug and gene delivery [42,43]. Among pullulan derivatives, hydrogels based on carboxymethyl pullulan (CMP) were prepared mainly using a crosslinking reaction with different diamines and dihydrazide [44] or 1-ethyl-3-(3-dimethylaminopropyl) carbodiimide/N-hydroxysuccinimide (EDC/NHS) [45]. Mocanu et al. [46] used two difunctional Jeffamines, ED-600 and ED-2003, for the synthesis of hydrogels based on CMP with amphiphilic and thermosensitive characteristics. Recently, an injectable enzymatically crosslinked CMP/Chondroitin sulfate hydrogel was reported as an appropriate candidate for cartilage tissue engineering [47].

Herein, to our knowledge, we report for the first time the synthesis of an injectable CMP-poloxamer copolymer. The effect of the polymer concentration on the gelation behavior was analyzed by different methods. The influence of the rest temperature before tests on the gelation starting time was also analyzed. Finally, various physico-chemical properties of the new copolymer related to its potential application as a drug delivery system were investigated.

## 2. Materials and Methods

### 2.1. Materials

Pullulan (P) (M_w_ = 200 KDa) was purchased from Hayashibara Lab. Ltd. (Okoyama, Japan). Monochloroacetic acid (MCA), sodium borohydride (NaBH_4_), poloxamer 407 (Plx), 4-nitrophenyl chloroformate (4-NF), ethylenediamine (EDA), 2-ethoxy-1-ethoxycarbonyl-1,2-dihydroquinoline (EEDQ) and amoxicillin (AM) were purchased from Sigma-Aldrich Co. (St. Louis, MO, USA). Isopropyl alcohol, dichloromethane and ethyl ether were supplied from Sigma-Aldrich Co. and used as received.

### 2.2. Synthesis of Monoamine Derivative of Poloxamer (MAPlx)

A monoamine poloxamer (MAPlx) derivative was prepared in two steps according to the method described by Cho et al. [34]. In the first step, 4-nitrophenyl formate-derivatized poloxamer was synthesized by the reaction in dichloromethane of poloxamer (1 g; 0.397 mmol) with an excess of 1.25:1 equivalents of 4-nitrophenyl chloroformate, at room temperature, for 4 h. The intermediate was recovered by precipitation in petroleum ether and subsequently washed with petroleum ether. The product was dried in a vacuum for 24 h. In the second step, the intermediate (0.34 mmol) was reacted with an excess of 3:1 equivalents of ethylenediamine. The reaction took place at room temperature in dichloromethane for 24 h. Finally, the reaction mixture was precipitated in petroleum ether and washed with ethyl ether. The dry precipitate was redissolved in distilled water and dialyzed against distilled water (molecular weight cut-off 2000 g/mol) for 5 days. The purified product was recovered by lyophilization.

### 2.3. Synthesis of Carboxymethyl Pullulan (CMP)

In a round-bottom flask with two necks, 20 g of P and 0.060 g NaBH_4_ were dissolved in 35 mL of pure water. To the resulting solution, 40 mL NaOH aqueous solution 38% (*w*/*v*) and 35 g of MCA (MCA/OH molar ratio 1/1) were added sequentially at 50 °C as follows: (i) 20 mL of NaOH aqueous solution and 17.5 g of MCA were added and the stirring continued at 50 °C for 1 h; (ii) 10 mL of NaOH aqueous solution and 8.75 g of MCA were added and the stirring continued for an additional 30 min; (iii) 10 mL of NaOH aqueous solution and 8.75 g of MCA were added, and the resulting solution was stirred at 50 °C for 24 h. After cooling at room temperature, the solution was dialyzed against distilled water (dialysis bag from Medicell International, England; molecular weight cut-off 12,000 g/mol) for 7 days, until the presence of chlorine ions in the washing water was no longer detected (by checking with AgNO_3_ solution). The final product was recovered by freeze-drying (−57 °C, 5.5 × 10^−4^ mbar) using a lyophilizer ALPHA 1–2 LD Christ, Germany.

### 2.4. Synthesis of Grafted Copolymer (Plx-g-CMP)

Over an aqueous solution of CMP (1%, *w*/*v*; 1.89 meq. COOH groups) acidified to pH = 3 with 0.1 N HCl, 0.12 mmol of EEDQ in methanol (1.5 mL) were dropwise added. The activated polysaccharide solution was then very slowly dripped at room temperature, over an aqueous solution of MAPlx (6%, *w*/*v*; 0.18 mmol). The reaction took place at room temperature for 24 h. Finally, the reaction mixture was dialyzed successively, firstly against distilled water/methanol mixture (4/1, *v*/*v*) for 3 days and then distilled water for 4 days (dialysis bag from Medicell International, England; molecular weight cut-off 12,000 g/mol). The final product was recovered by lyophilization for 48 h at −57 °C and 5.5 × 10^−4^ mbar.

### 2.5. Structural Characterization

^1^H nuclear magnetic resonance (^1^H−NMR) spectra were carried out on a Bruker Avance DRX 400 NMR spectrometer (Rheinstetten, Germany) at 25 °C, in D_2_O for CMP and D_2_O/0.7% NaOH aqueous solution for poloxamer-*graft*-carboxymethyl pullulan (Plx-*g*-CMP).

Fourier transform infrared spectroscopy (FT-IR) was performed to confirm the composition of copolymers. The FT-IR spectra of copolymers were recorded in their solid (lyophilized) state pelletized with KBr, on a FT-IR Vertex 70 spectrometer (Bruker, Vienna, Austria) in the range of 4000–400 cm^−1^ at a resolution of 4 cm^−1^.

The substitution degree (*DS*) with carboxymethyl groups of CMP was determined by conductometric titration of CMP with a 0.1 N HCl aqueous solution, using a conductometer CMD 210 (Radiometer, Copenhagen, Denmark) equipped with a CDC 865 cell. The carboxymethyl groups content (*C*) of the polymer was calculated with Equation (1):

(1) $$C\left(meq./g\right)\;=\;\frac{V_{HCl}\times c}{m}$$

where *V_HCl_* represents the volume of HCl aqueous solution determined from the conductometric titration curve, *c* is the concentration of HCl solution (mol/L) and *m* represents the weight of the dried sample (*g*) used in titration. *DS* (number of carboxymethyl groups per anhydroglucose unit (UG) in pullulan) was determined with Equation (2):

(2) $$DS=\frac{162\times C}{162+80\times C}$$

where *C* represents the content of carboxymethyl groups in equivalents/gram of polymer, 80 is the molecular mass of the carboxymethyl radical and 162 represents the molecular mass of anhydroglycosidic unit.

The number-average molar mass (*M*n) of CMP was measured with a Shimadzu HPLC System with a refractive index detector, a 10 mm × 250 mm column packed with 5 μm mixed bed linear Jordi DVB Glucose gel (Grace Davison Discovery ScienceTM). Polymer solution (0.5 mg/mL, 20 μL) was injected into column and elution was performed with aqueous 1 N NaOH (0.5 mL/min) at 25 °C. Dextran standards were used for calibration.

### 2.6. Hydrogel Characterization

#### 2.6.1. Thermosensitive Sol–Gel Transition Behavior

The thermosensitive sol–gel transition of Plx and Plx-*g*-CMP solutions was measured by the tube inverse method [34]. Basically, 1 mL of a sample at concentrations ranging from 18 to 11% (*w*/*v*) in phosphate buffer (PB) with pH = 7.4 was prepared and the gelation temperature was visually checked by maintaining the sample at different temperatures (20, 21, 22, 26 and 33 °C) for 15 min in a water bath. The gelation temperature was determined as the temperature at which the solution became gel and did not flow by the vial inverting for 60 s. Each value represents the average with the standard deviation (SD) obtained from three measurements.

#### 2.6.2. Rheological Analysis

A rheological analysis of the Plx-*g*-CMP samples was carried out by using an MCR 302 Anton Paar rheometer (Gratz, Austria) with plane-plane geometry (the diameter of the upper plate being 50 mm, gap of 500 μm). The rheometer was equipped with a Peltier device ensuring a rigorous temperature control. In order to prevent the water evaporation, an anti-evaporation device was used to create a saturated atmosphere of solvent in the vicinity of the sample.

In the oscillatory deformation tests, the following viscoelastic parameters were determined: the elastic modulus, *G′*, as a measure of the stored deformation energy; the viscous modulus, *G″*, as a measure of the energy dissipated during one cycle of oscillatory deformation and the loss tangent, tan *δ*, as a measure of the degree of viscoelasticity characteristic to the sample in the investigated conditions (it is calculated as a ratio between the loss energy to the accumulated energy in a cyclic deformation). The dynamic oscillatory behavior of the Plx-*g*-CMP samples with different concentrations (13, 22 and 30%, *w*/*v*) was investigated in the linear range of viscoelasticity.

The gelation process was investigated at 37 °C by using samples kept at 4 °C before the starting of the experimental tests. Approximately 2 mL of homogeneous solution were poured on the lower plate of the rheometer thermostated at 4 °C. When the temperature of 37 °C was reached, the viscoelastic parameters were followed as a function of time at a constant oscillation frequency (*ω*) of 1 rad/s and strain amplitude (*γ*) of 1%. Similar tests were performed when the storing temperature was 20 °C. The gelation was also followed in temperature sweep experiments at a heating rate of 0.5 °C/min, the starting temperature being 4 °C and the maximum temperature reached being 60 °C. Small-amplitude oscillatory shear experiments (*γ* = 1%) were carried out at a constant temperature of 37 °C, for oscillation frequencies between 0.1 rad/s and 100 rad/s.

The viscoelastic moduli, *G′* and *G″*, were measured as a function of time in self-healing tests when *ω* was set constant at 1 rad/s and *γ* was successively settled at low and high strain amplitude values, 1% and 100%, respectively.

### 2.7. Drug Loading and In Vitro Release Studies

Drug incorporation in hydrogel was performed by dissolving 40 mg of amoxicillin (AM) in 10 mL Plx-*g*-CMP solution (in PB at room temperature) with two concentrations (13 and 22%, *w*/*v*). Then, samples of 1 mL Plx-*g*-CMP solution with a solubilized drug were prelevated and placed in separate vials for gelling at 37 °C. The release studies were performed according to Cho et al. (2003) [34]. The vials were filled with 10 mL of PB and the temperature and stirring rate were maintained at 37 °C and 50 rpm, respectively. Samples of the receiving buffer (1 mL) were withdrawn at different time intervals and the drug content was determined by spectrophotometric analysis using an Evolution 201 UV spectrophotometer (Thermo Fisher Scientific Inc., Madison, WI, USA) based on a standard curve of the pure drug in PB at 228 nm (y = 0.0219x; R^2^ = 0.9993). The same volume of the fresh receiving buffer was added to replace the volume of the withdrawn samples.

The release kinetics of AM from Plx-*g*-CMP hydrogels were analyzed by the following mathematical models:

Zero-order:

(3) $$\frac{M_{t}}{M_{\infty }}=k_{0}\;t$$

Higuchi:

(4) $$\frac{M_{t}}{M_{\infty }}=k_{H}t^{1/2}$$

Korsmeyer–Peppas:

(5) $$\frac{M_{t}}{M_{\infty }}=kt^{n}$$

Peppas–Sahlin:

(6) $$\frac{M_{t}}{M_{\infty }}=k_{1}t^{m}+k_{2}t^{m}$$

where *M_t_*/*M*_∞_ is the fraction of drug released at time *t*, *k*_0_ and *k*_H_ represent the zero-order and Higuchi release kinetic constants, respectively, *k* is a constant defined by the structural and geometric characteristic of the dosage form and *n* is the release exponent indicative for the drug release mechanism [48]. For cylindrical geometries, *n* = 0.45 suggests Fickian diffusion, *n* = 0.9 refers to a non-Fickian with the characteristic of zero-order release, if 0.45 < *n* < 0.9 the transport process is anomalous with a superposition of diffusion and swelling controlled drug release. If *n* < 0.45, a pseudo-Fickian diffusion with a slow release occurs [49]. In the Peppas–Sahlin model [50], *k*_1_ and *k*_2_ are the kinetic constants related to the Fickian and non-Fickian diffusional contributions, respectively.

For establishing the model that statistically best represents the drug release mechanism, the Akaike number, Akaike Information Criterion (*AIC*), [51] defined by Equation (7) was used in the discrimination analysis.

(7) $$AIC=N\times \ln\left(SSR\right)\;+\;2\times p$$

where *N* is the number of experimental data, *SSR* is the square residual sum and *p* is the number of parameters in the model.

## 3. Results and Discussion

### 3.1. Synthesis and Characterization of Plx-g-CMP

**Scheme 1:**
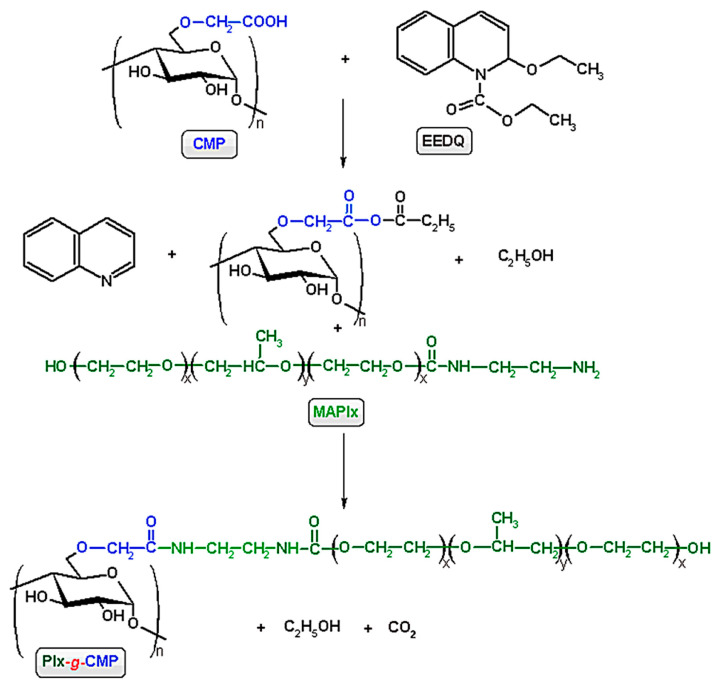
Synthesis route of poloxamer-*graft*-carboxymethyl pullulan copolymer.

The grafted copolymer (Plx-*g*-CMP) was prepared by the coupling reaction between the amino groups of functionalized poloxamer and carboxylic groups present on the pullulan chains. The reaction occurred with the formation of an amide bond (Scheme 1) in the presence of 2-ethoxy-1-ethoxycarbonyl-1,2-dihydroquinoline (EEDQ). It is well known that EEDQ is a specific coupling reagent for carboxyl groups widely used in the formation of peptide bonds [52].

**Scheme 2:**
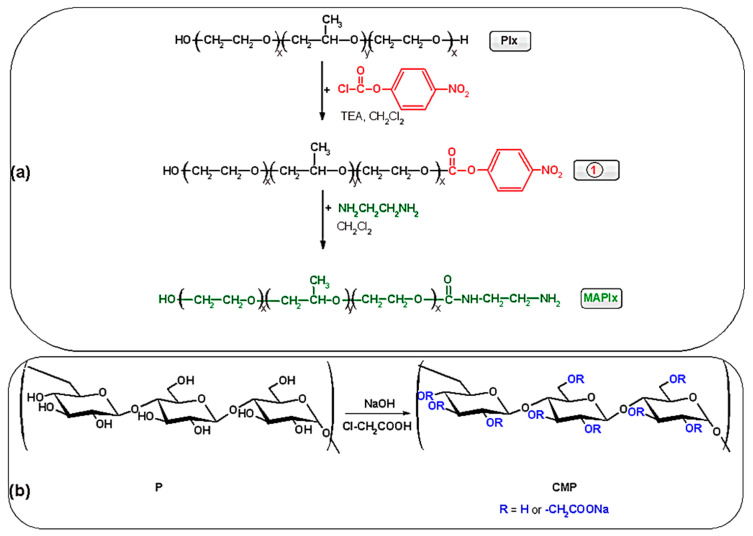
Synthesis route of monoamine derivative of poloxamer (MAPlx) (**a**) and carboxymethyl pullulan (**b**).

The synthesis of the monoamine derivative of poloxamer was performed according to the method described by Cho et al. (2003) [34] (Scheme 2a) and CMP was obtained by the carboxymethylation reaction of pullulan, using sodium chloroacetate as a carboxymethylating agent (Scheme 2b), according to Asmarandei et al. [53].

**Figure 1:**
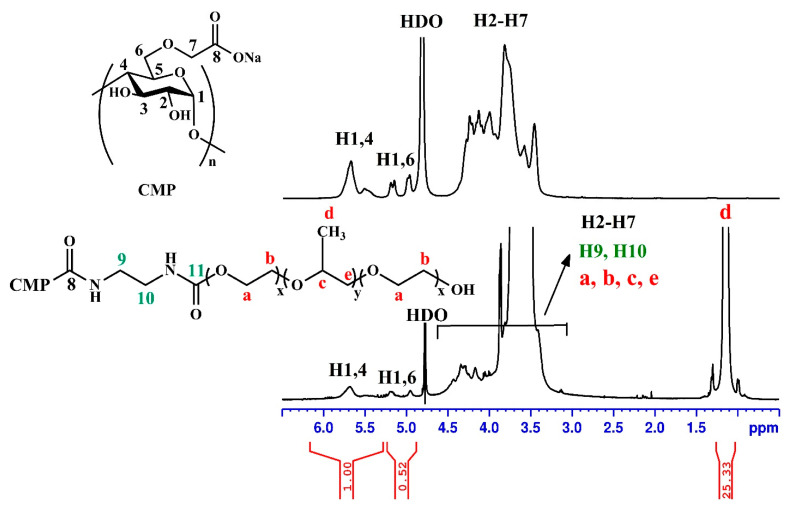
^1^H NMR spectra of CMP and Plx-*g*-CMP.

The ^1^H NMR spectrum of CMP in D_2_O confirmed that the carboxymethylation reaction of the pullulan occurred, by the presence of the characteristic signal of methylene protons at 4.16 ppm (Figure 1). Since this signal overlaps with those specific to the protons in the glucoside units, the determination of the substitution degree (*DS*) was not possible by this method. Therefore, the *DS* of pullulan with carboxymethyl groups was determined by the titration method with 0.1 N HCl solution and found to be 0.87 (29/100 sugar units) [54] (data not shown). The *M*n value of CMP estimated by the gel permeation chromatography was found to be 1.94 × 10^4^ g/mol (*M*w/*M*n = 2.3), close to the *M*n of pullulan, which is an indication that the carboxymethylation reaction took place without the chain cleavage.

The ^1^H NMR spectrum of the grafted copolymer (Figure 1) shows the presence of the typical signal corresponding to methyl groups in the propylene oxide units of the poloxamer at 1.14 ppm. The other signals from methylene and methyne protons in PPO and PEO units of Plx appeared together with the specific protons of the anhydroglucosidic unit of CMP in the region between 3.0 and 4.6 ppm. Additionally, the (1→6) and (1→4) anomeric protons at 5.67 and 4.95 ppm and the hydrogen atoms H2-H7 from the glucosidic unit of CMP are present. Based on this spectrum, the composition of the polymer was determined by reporting the integral value of C*H*_3_ protons at 1.14 ppm to that of *H*-1 protons of CMP observed at 4.95 and 5.67 ppm (Equation (8)).

(8) $$DS\;\left(\%\right)=\frac{I_{CH3}/3\times y}{I_{H\left(1,4\right)}+I_{H\left(1,6\right)}}\times 100$$

where *y* = 56 (considering the poloxamer composition (PEO)_*x*_(PPO)_*y*_(PEO)_*x*_ (*x* = 101, *y* = 56).

The weight content of 83.88% Plx of the grafted polymer was obtained, equivalent to a *DS* equal with 10 mol% (mol of Plx/100 anhydroglycosidic unit of CMP) and a carboxymethyl groups content of 0.59 mmol/g. The average number of graft chains/100 sugar units of Plx-*g*-CMP calculated from ^1^H NMR spectrum was 2.9 and the *M*n value was 57.7 × 10^4^ g/mol.

**Figure 2:**
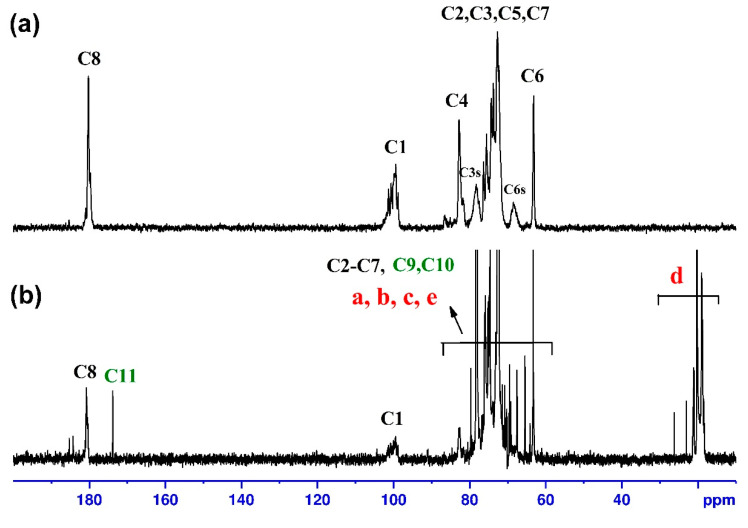
^13^C NMR spectra of CMP (**a**) and Plx-*g*-CMP (**b**).

The evidence for a successful grafting of Plx to carboxymethyl groups of CMP via amide linkages was also revealed in the ^13^C NMR spectrum of Plx-*g*-CMP (Figure 2) where, in the region between 170 and 185 ppm, three distinct signals are present. Besides the signal at 180 ppm belonging to C8 in CMP component, the peak at 174 ppm could be attributed to C11 (carbonyl in amide group of Plx) and the small signals at 185 ppm corresponded to C=O in unprotonated carboxymethyl groups of CMP.

**Figure 3:**
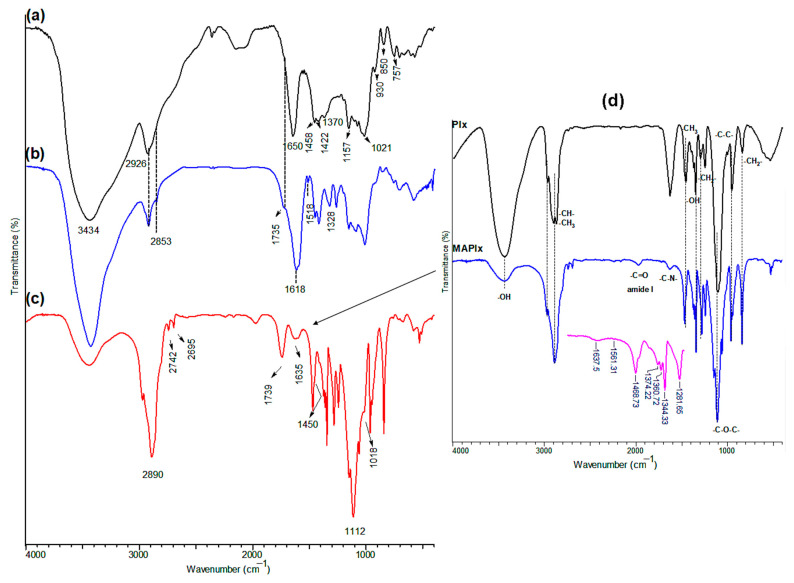
FT-IR spectra of pullulan (**a**), CMP (**b**) and Plx-*g*-CMP (**c**); for comparison, the FT-IR spectra of Plx and MAPlx are presented (**d**).

The FT-IR spectra of native pullulan, CMP derivative and Plx-*g*-CMP copolymer are presented in Figure 3. The characteristic absorption bands of pullulan (Figure 3a) are present in the FT-IR spectrum of CMP (Figure 3b). Moreover, the appearance of new bands specific to newly introduced carboxymethyl groups confirms the successful achievement of the carboxymethylation reaction. Most importantly, the peak at 1735 cm^−1^ illustrates the formation of an ester bond and the absorption bands observed at 1618 and 1423 cm^−1^ highlight the stretching and bending vibrations of the carbonyl (C=O) in the carboxymethyl groups. The other bands were assigned as follows: 3435 cm^−1^ (O-H stretching vibration), 2924 and 2853 cm^−1^ (C-H stretching vibration of methylene groups), 1328 cm^−1^ (C-O stretching), 1157 cm^−1^ and 1095 cm^−1^ (C-O-C vibration and C-O stretching bands, respectively) [55], 858 cm^−1^ corresponding to α-configuration of α-D-glucopyranoside units [56] and those at 765 and 930 cm^−1^ indicated the α(1–4) and α(1–6) linkages, respectively [57]. The peaks at around 850 and 765 cm^−1^ show the pullulan’s ^4^C_1_ chair conformation.

The FT-IR spectrum of Plx-*g*-CMP (Figure 3c) shows both the specific absorption bands of CMP and Plx (2890 cm^−1^ (C-H stretching aliphatic), 1385 cm^−1^ (in-plane O-H bend) and 1112 cm^−1^ (C-O-stretch) [58]) (see Figure 3d). In addition, a shift of the ester absorption band from 1735 to 1739 cm^−1^ is observed, as well as the appearance of absorption bands specific to the amide bond (1635 cm^−1^ (C=O, amide I), 1554 cm^−1^ (coupling NH and CN, amide II) and 1450 cm^−1^ (C-N stretch, amide III)), which demonstrates the coupling of poloxamer to the carboxymethyl pullulan chain through an amide bond.

The density of the grafted polymer measured by the pycnometry method [59] in tetrahydrofuran (THF) at 25 °C, is 1.336 g/cm^3^ and it remains soluble in water and polar solvents (dimethylfuran (DMF), dimethyl sulfoxide (DMSO), etc.), and insoluble in THF, ethyl ether, and chloroform.

### 3.2. Gelation Temperature

**Figure 4:**
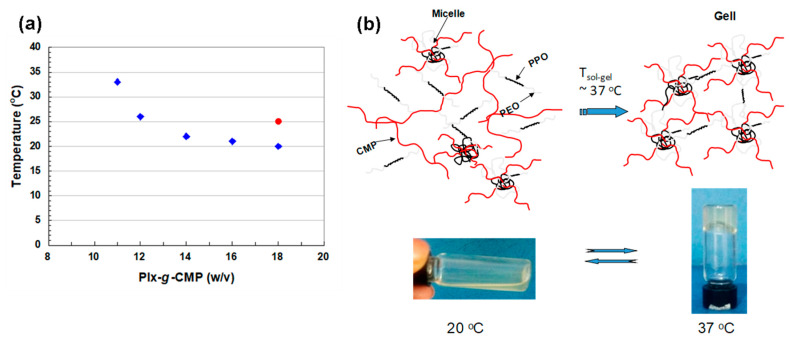
Influence of the polymer concentration on the gelation temperature of Plx (•) and Plx-*g*-CMP (◆) in phosphate buffer 0.05 M at pH = 7.4 (**a**). Schematic representation of sol–gel transition of the Plx-*g*-CMP solution (**b**).

The influence of Plx-*g*-CMP concentration on the gelation temperature is shown in Figure 4a. It is clearly evident that the gelation temperature decreases with an increasing copolymer concentration: from 33 °C at a concentration of 11% (*w*/*v*) to 20 °C at a concentration of 18% (*w*/*v*). It must be underlined that the gelation temperature of Plx-*g*-CMP (20 °C) is lower than that of poloxamer (25 °C) at the same concentration (18%, *w*/*v*). Furthermore, the sol–gel transition of grafted polymer occurs until a concentration of 11% (*w*/*v*), while the gelation of poloxamer does not occur at lower concentrations. These observations certify that Plx-*g*-CMP forms supplementary crosslinking junctions (i.e., hydrogen bonds); in fact, the Plx-*g*-CMP is currently a copolymer with a high molecular weight, possessing carboxylic groups able to interact intra- and intramolecularly with free OH groups present on the polymeric chains. Moreover, under simulated physiological conditions at pH = 7.4, the polymer chains exist in their expanded state and the environment of the PPO segments become more polar, forcing the PPO to aggregate at lower temperatures (Figure 4b).

### 3.3. Rheological Behavior

**Figure 5:**
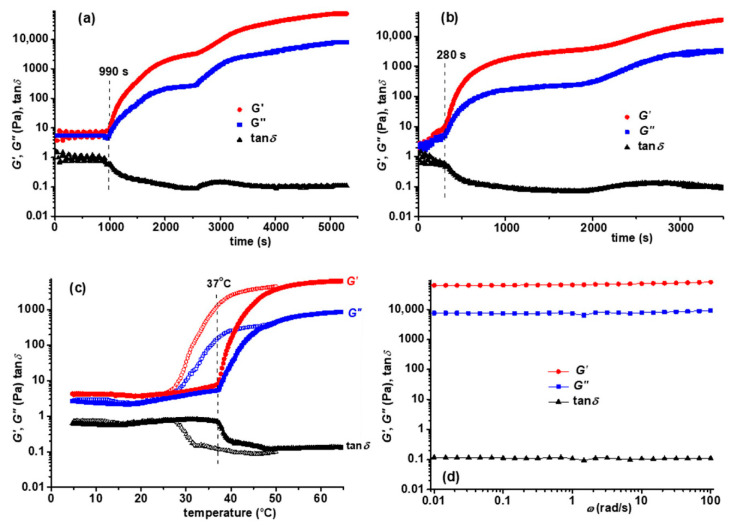
The evolution of the viscoelastic parameters in time for 13% (*w*/*v*) Plx-*g*-CMP after 24 h of rest at (**a**) 4 °C; (**b**) 20 °C (ω = 1 rad/s, *γ* = 1%), raising the sample temperature at 37 °C inside of the rheometer; as a function of temperature at increasing (full symbols) and decreasing the temperature (empty symbols) after 24 h of rest at 4 °C (heating/cooling rate of 0.5 °C/min, ω = 1 rad/s, *γ* = 1%) (**c**); as a function of the oscillation frequency after 2 h of rest at 37 °C for the sample previously kept at 4 °C (*γ* = 1%) (**d**).

Figure 5 presents the gelation kinetics of Plx-*g*-CMP at a concentration of 13% (*w*/*v*) when the sample is heated in situ at the physiological temperature. Firstly, the sample was introduced into the rheometer geometry at 4 °C (Figure 5a) or 20 °C (Figure 5b), heated at 37 °C and then the evolution of the viscoelastic parameters was followed in time at a constant oscillation frequency (1 rad/s) and strain amplitude (1%). Depending on the rest temperature before the test, the gelation started after 990 s or 280 s, when *G′* and *G″* increase very fast, more than two decades, and then the viscoelastic parameters change more slowly in time as the tridimensional structure evolves (tan *δ* < 1) to a stationary state that is independent of the rest temperature or Plx-*g*-CMP concentration. For higher concentrations (22 and 30%, *w*/*v*), the gelation time is tenths of seconds for samples kept at 4 °C, whereas for samples kept at 20 °C, the gelation occurs nearly instantaneous when the temperature is raised to 37 °C, making these samples not appropriate for an injectable hydrogel. In the case of a concentration of 13% (*w*/*v*), a gelation time of 280 s obtained for the sample stored at 20 °C can be considered a too short a time to develop a network structure; moreover, 990 s could be an optimum gelation time for an injectable hydrogel. Another observation is the fact that for all samples it was observed that there are two steps during the gelation process (as, for example, after 990 s and 2500 s in Figure 5a or 280 s and 2000 s in Figure 5b), which is probably due to the competition between different types of interactions (hydrogen bonds, hydrophobic interactions) that develop in time.

For samples kept 24 h at 4 °C, the viscoelastic parameters were also followed as a function of temperature for a heating rate of 0.5 °C/min, ω = 1 rad/s, *γ* = 1%. An increase of the viscoelastic moduli was registered at the gelation temperature that is located around 37 °C for 13% Plx-*g*-CMP (Figure 5c) and below 20 °C for higher concentrations. In the gel state, tan δ ≅ 0.08–0.1 regardless of the polymer concentration. Another observation concerns the behavior at a decreasing temperature, when the sample is submitted to cooling with a rate of 0.5 °C/min. Between 50 °C and 28 °C, there is a delay in the answer to the thermal stimulus, whereas the complete and fast structure recovery was observed at low temperatures (below 28 °C), when the hydrophobic interactions diminished considerably.

As the network structure is well formed at 37 °C (after about 2 h of rest at 37 °C), the viscoelastic parameters are independent on the oscillation frequency (*γ* = 1%) as it is shown in Figure 5d; *G′* > *G″* and tan *δ* ≅ 0.1. The gel strength is improved in this case, *G′* is about ten times higher as compared with the values registered in the temperature sweep tests at temperatures above 50 °C. The slower dynamics of macromolecules at 37 °C allows for the manifestation of interactions that favor the gelation in time, as it was shown in Figure 5a,b.

For the injectable hydrogels in situ formed in the human body, the self-repair ability represents an important characteristic [60,61]. The recovery of mechanical performances is a great challenge for biomaterials, and an irreversible deformation can induce local heating or the loss of functionality, limiting the life service of the gels and finally causing an infection [62].

**Figure 6:**
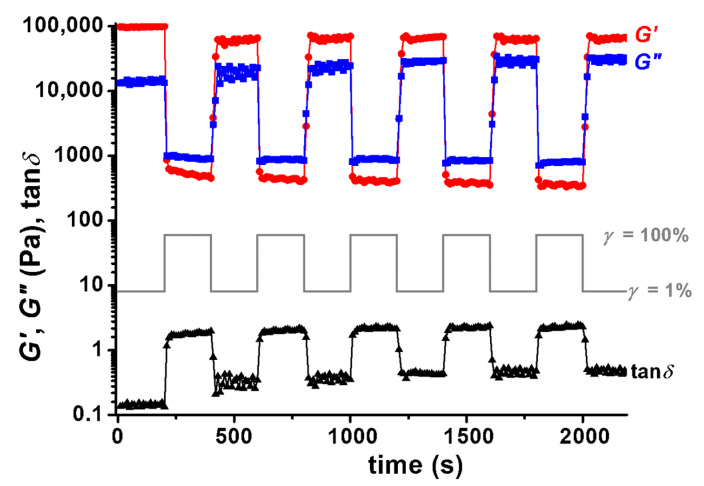
Self-healing test for the hydrogel containing 13% (*w*/*v*) Plx-*g*-CMP by following the evolution of the viscoelastic parameters in time, when two successive levels of deformations were applied each for 200 s: 1% and 100% (37 °C, 1 rad/s).

In order to evaluate the self-healing behavior, the thixotropy was carefully investigated for the Plx-*g*-CMP hydrogels at 37 °C following the time dependence of the viscoelastic parameters (*G′*, *G″* and tan *δ*) when two successive levels of deformations were alternatively applied: 1% and 100% (Figure 6).

When the sample is submitted to a large oscillatory deformation (*γ* = 100%), *G′* and *G″* decrease and the network structure is lost (tan *δ* > 1) and the sample fluidity increases. By switching the deformation from a large strain value of 100% to a small one of 1%, the network structure is recovered in a few seconds. The rest structure is not completely recovered after the first cycle of low and high deformation (the *G’* value after the first cycle is about 65% from its rest value). The test was repeated five times and it was observed that the healing process is reversible for the next four cycles of deformation; the sample is able to recover its network structure after the successive action of the external forces (tan *δ* is about 0.14 at rest and it increases slowly between 0.2 and 0.4 after applying one to five cycles of deformation, respectively).

### 3.4. Drug Loading and In Vitro Release Studies

Amoxicillin (AM) was chosen as a model drug because it is frequently used to treat degenerative disc disease or other related disorders; moreover, the injectable polymers loaded with AM can be used for the treatment of such diseases. The incorporation of AM in Plx-*g*-CMP hydrogel was performed by solubilization of the drug in the polymeric solution at a low temperature followed by its gelation at the temperature of 37 °C. In this way, the entire amount of drug is retained in the hydrogel, having a uniform distribution within the three-dimensional matrix. In fact, the polymer has an anionic charge and hydrophobic pendant chains, therefore, it can interact with both charged and hydrophobic drugs.

**Figure 7:**
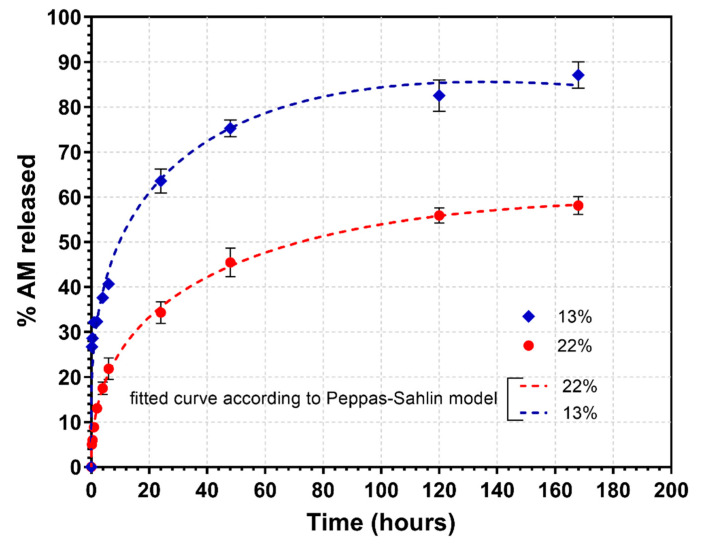
In vitro release profiles of AM from Plx-*g*-CMP hydrogels obtained at two concentrations (13% and 22%, *w*/*v*).

The release studies of the AM from the hydrogel obtained at two polymer concentrations (13 and 22%, *w*/*v*) were performed under simulated physiological conditions (PB at pH = 7.4) (Figure 7). A gradual release of AM from both hydrogels over a period of about 168 h can be observed. Within the first 6 h, 41% of the drug is released from Plx-*g*-CMP hydrogel obtained at a concentration of 13% (*w*/*v*), which is twice than that of the amount of drug released from the hydrogel obtained at a concentration of 22% (*w*/*v*). For the next 48 h, the release profiles follow almost the same pattern but the difference is lower. From this point and until the end of the study, the profile of the release curves follows an approximately parallel trajectory, suggesting that the diffusion of the drug is the rate-determining step. However, the diffusion of AM from the hydrogel to the release fluid should be controlled both by steric interactions with the polymer network and by the possible electrostatic interactions between an opposite charged drug/CMC. As long as release studies are performed in a phosphate buffer with a certain ionic strength (50 mM), the electrostatic interactions between the drug and carboxylic groups of CMP could be shielded by the presence of competing ions. As a result, the rate of drug diffusion will be controlled only by the steric interactions between the drug and the polymer network. Evidently, the denser the network, the higher the probability of intermolecular interactions and the lower the release rate.

**Figure 8:**
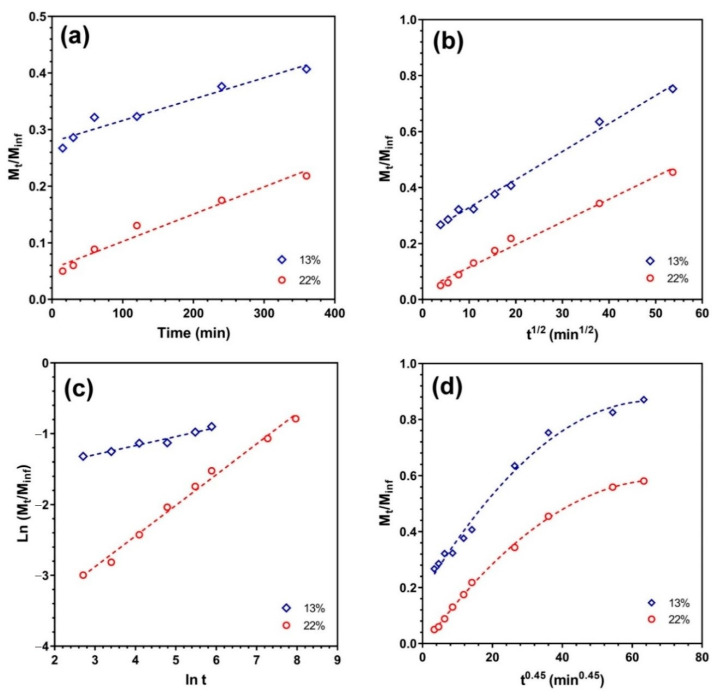
Release dynamics of AM from loaded Plx-*g*-CMP hydrogels in PB (pH 7.4) at 37 °C: plots of zero-order (**a**), Higuchi (**b**), Ritger–Peppas (**c**) and Peppas–Sahlin (**d**) models.

In order to further understand the in vitro drug release mechanism of AM, the experimental data were analyzed using the mathematical models described by Equations (3)–(6) (Figure 8). The results (Table 1) revealed that the in vitro release profile of AM in PB (pH 7.4) followed the Higuchi equation (low AIC value), and according to the Ritger–Peppas model, the drug release mechanism pointed out the prevalence of the diffusional mechanistic phenomena, since the values of the diffusional exponent *n* obtained were lower or close to the standard value for declaring Fickian release behavior (*n* ≤ 0.45).

As shown in Table 1, the Peppas–Sahlin model possesses a lower AIC value than that of the Higuchi and Ritger–Peppas models, which indicates that the former was the more appropriate one to describe the release data than the latter (see Figure 7). These observations indicate that the drug release mechanism was a result of the contribution of Fickian diffusion and polymer chain relaxation. However, a negative value of *k*_2_ was obtained, which means that the relaxation process has a negligible effect on the AM release compared to the Fickian diffusion.

For a hydrogel matrix that contains a molecularly dispersed diffusing drug, the apparent diffusion coefficient may be calculated by using the 1D unsteady-state form of Fick’s second law of diffusion, which for small values of time (*t* < 60%) is given by Equation (9) [36]:

(9) $$\frac{M_{t}}{M_{\infty }}\;=\;4\;{\left(\frac{D_{E}\;t}{\pi \;H^{2}}\right)}^{0.5}$$

where *M_t_* and *M*_∞_ are the total mass of the diffusion compounds released from the layer after time *t* and infinite time, respectively. Since for most systems the diffusion coefficient (*D_E_*) is dependent on drug concentration, as well as the concentration of the swelling agent (i.e., water), the slope of the plot of *M_t_*/*M*_inf_ as a function of *t*^0.5^ generates an average diffusion coefficient. The thickness of the hydrogel matrix (*H*) inside the vial was calculated from the volume of the Plx-*g*-CMP solution (i.e., 1 mL) and from the dimensions of the vial. *D*_E_ values obtained for the two concentrations of hydrogel from plotting *M_t_*/*M*_∞_ vs. *t*^0.5^ (Figure 8b) were 2.579 × 10^−7^ mm^2^/min for the 13% (*w*/*v*) sample and 1.692 × 10^−7^ mm^2^/min for the 22% (*w*/*v*) one. This reduction of the diffusion rate with the increase of the Plx-*g*-CMP concentration confirms our hypothesis that steric hindrances control the rate of drug release.

## 4. Conclusions

Poloxamer-*graft*-carboxymethyl pullulan (Plx-*g*-CMP) copolymer was successfully synthesized by the coupling reaction between carboxymethyl groups of pullulan and amine groups introduced on poloxamer. The grafted copolymer has the ability to form a gel under simulated physiological conditions (phosphate buffer at pH = 7.4 and 37 °C). The sol–gel transition temperature of the copolymer is lower (20 °C) than that of Plx (25 °C) at the same concentration (18%, *w*/*v*). Moreover, the sol–gel transition of the grafted copolymer occurs until a concentration of 11% (*w*/*v*), while the gelation of poloxamer 407 does not occur at concentrations lower than 18% (*w*/*v*). The rheological tests evidenced a high elasticity and ability of Plx-*g*-CMP to recover the structure after the removal of the applied force or other external stimuli (such as temperature).

The Plx-*g*-CMP hydrogel synthesized at two concentrations (13% and 22%, *w*/*v*) proved to be a suitable support for the sustained delivery of amoxicillin (taken as model drug).

Based on the physico-chemical and rheological properties, the copolymer is recommended as an appropriate material in cartilage tissue engineering. Further studies concerning its cytocompatibility and degradability need to be performed. Moreover, new investigations must be conducted to improve the gelation time and the hydrogel durability by variation of the number of grafted chains or by introducing a supplementary covalent crosslinking.

## Figures and Tables

**Table 1:** Release parameters corresponding to AM-loaded Plx-*g*-CMP hydrogels of 13% and 22% copolymer concentration.

Conc. (%, *w*/*v*)	Zero-Order			Higuchi			Ritger–Peppas				Peppas–Sahlin			
	*k*_0_ × 10^4^	R^2^	AIC	*k*_H_ × 10^2^	R^2^	AIC	*K* × 10^2^	*n*	R^2^	AIC	*k*_1_ × 10^2^	*k*_2_ × 10^4^	R^2^	AIC
13	3.77	0.941	−35.35	1.001	0.993	−56.36	18.68	0.13	0.958	−24.16	2.073	−1.56	0.991	−60.29
22	4.82	0.969	−36.41	0.810	0.986	−54.03	1.534	0.43	0.992	−30.93	1.72	−1.24	0.999	−84.96

## Data Availability

Data are available on request.
